# Binder-Free Electrode Based on ZnO Nanorods Directly Grown on Aluminum Substrate for High Performance Supercapacitors

**DOI:** 10.3390/nano10101979

**Published:** 2020-10-07

**Authors:** Faheem Ahmed, Ghzzai Almutairi, Bandar AlOtaibi, Shalendra Kumar, Nishat Arshi, Syed Ghazanfar Hussain, Ahmad Umar, Naushad Ahmad, Abdullah Aljaafari

**Affiliations:** 1 Department of Physics, College of Science, King Faisal University, P.O. Box-400, Al-Ahsa 31982, Saudi Arabia; sjagdish@kfu.edu.sa (S.K.); aaljaafari@kfu.edu.sa (A.A.); 2 National Center for Energy Storage Technologies, King Abdulaziz City for Science and Technology (KACST), Riyadh 12354, Saudi Arabia; bmalotaibi@kacst.edu.sa; 3 Department of Basic Sciences, Preparatory Year Deanship, King Faisal University, Al-Ahsa 31982, Saudi Arabia; nshastri@kfu.edu.sa; 4 Department of Chemistry, Faculty of Science and Arts, NajranUniversity, Najran 11001, Saudi Arabia; ahmadumar786@gmail.com; 5 Promising Centre for Sensors and Electronic Devices (PCSED), Najran University, Najran 11001, Saudi Arabia; 6 Department of Chemistry, College of Science, King Saud University, Riyadh 11451, Saudi Arabia; anaushad@ksu.edu.sa

**Keywords:** ZnO, binder-free electrode, X-ray diffraction, supercapacitors, nanorods

## Abstract

Herein, for the first time, the growth of ZnO nanorods directly on aluminum (Al) substrate via a low temperature (80 °C) wet chemical method, and used as binder-free electrode for supercapacitors were reported. XRD pattern and HRTEM images showed that high crystalline nanorods grown on Al substrate with c-axis orientation. Morphological studies revealed that the nanorods possessed well defined hexagon phase with length and diameter of ~2 µm and 100–180 nm, respectively. Raman spectrum of ZnO nanorods showed that the characteristic E_2H_ mode corresponds to the vibration associated with the oxygen atoms of ZnO. The optical properties of ZnO nanorods studied using Room-temperature PL spectra revealed a near-band-edge (NBE) peak at ~388 nm emission and deep level (DLE) at ~507 nm. Electrochemical measurements showed that ZnO nanorods on Al substrate exhibited remarkably enhanced performance as electrode for supercapacitors with a value of specific capacitance of 394 F g^−1^ measured with scan rate of 20 mV s^−1^. This unique nanorods structures also exhibited excellent stability of >98% capacitance retention for 1000 cycles that were measured at 1A g^−1^. The presented easy and cost-effective method might open up the possibility for the mass production of binder-free electrodes for efficient electrochemical energy storage devices.

## 1. Introduction

In recent years, among the different energy storage systems, supercapacitors have gained the significant interest of researchers and scientific community due to their superior power density, longer life cycle, and cost effective maintenance as compared with the batteries/fuel cells, while it showed better energy density than conventional capacitors [1,2]. These supercapacitors have been potentially used in various applications, including hybrid electric vehicles, solar power plants, and portable consumer electronics [3,4]. Furthermore, the utilization of cost-effective and non-toxic electrode materials used in supercapacitors made them a suitable choice for consumers with environmentally friendly features [3,4,5,6,7,8]. Ideal capacitive materials, such as carbon nanostructure, have shown promising results with excellent cycle life. In a report, Xiao et al. [9] inserted nitrogen into graphene using nitric acid through thermal treatment at 500 °C, which showed a capacitance value of 370 F g^−1^ at 1 A g^−1^ in 6 M NaOH. On the other hand, in general, a pseudocapacitor consists of a transition metal oxide, including RuO_2_ [10], MnO_2_ [11], V_2_O_5_ [12], or ZnO [13]. These oxides contain different valence states in which the charges can be stored either by physical adsorption or by performing reversible faradic charge-transfer reaction taking place on the surface of electrode. Thus, their relatively fast and reversible faradic redox reactions made the pseudocapacitor a promising device with high power and high energy density. Among the above-mentioned metal oxides, due to its excellent features of high quasi-metallic conductivity and high capacitance, RuO_2_ has attracted enormous interest as a suitable material for electrode [14]. Although, high cost, limited availability, and the environmental safety problems make this material unsuitable. Therefore, inexpensive transition metal oxides materials based electrodes with high energy density as a possible substitute for RuO_2_ are needed.

Recently, Zinc oxide (ZnO) with direct bandgap energy of 3.37 eV has been gained enormous interestdue to cost effectiveness and excellent electrochemical properties [15,16]. Several reports have been presented on ZnO based electrodes as a suitable candidate for supercapacitor. Chen et al. showed that ZnO nanorods coated with MnO_2_ resulted to a specific capacitance value of 222 F g^−1^ at 25 mV s^−1^ [17]. A value of specific capacitance of 62.2 F g^−1^ obtained at 0.5 Ag^−1^ had been reported for ZnO/graphene nanosheets [18]. In another work, A ZnO/graphene nanocomposites that were synthesized by using microwave methods showed a value of specific capacitance of 146 F g^−1^ [19]. In case of carbon nanotube–ZnO nanocomposites electrodes, Zhang et al. [20] reported a maximum specific capacitance of 323.9 F g^−1^. On the other hand, graphene–ZnO composite films exhibited a specific capacitance of 11.3 F g^−1^ [21]. A composite containing ZnO/carbon that was reported by Jayalakshmi et al. [22] demonstrated a value of specific capacitance of 21.7 F g^−1^. In another report, composite electrodes containing activated carbon nanofiber and ZnO that was prepared by Kim et al. [23] reported a specific capacitance of 178.2 F g^−1^. It is clear that all of these works mentioned above were mainly focused on ZnO based composites for supercapacitors. However, less attention has been given on the use of ZnO as an electrode material, thus there is a lack of research reported on ZnO for supercapacitors. He et al. [24] demonstrated the growth of ZnO nanocones that were obtained by chemically etching ZnO nanowires (NWs) and obtained a value of 378.5 F g^−1^ for ZnO nanocones and 191.5 F g^−1^ for ZnO NWs, respectively. In another work [25], ZnO particles that were prepared by hydrothermal method with various precursors nitrate, acetate and chloride produced specific capacitance of 5.87 F g^−1^, 5.35 F g^−1^, and 4.14 F g^−1^, respectively. In recent work, Luo et al. [26] showed maximum capacitance value of 160.4 F g^−1^ for the electrodes that were based on ZnO tetrapods structures. Though the nanocomposites of ZnO and ZnO alone have been demonstrated to exhibit a comparable specific capacitance value [17,18,19,20,21,22,23,24,25,26], during charge and discharge cycling, a loosely adhered layer on the current collector might be risky and detached from the surface, leading to the poor performance of ZnO electrodes [27,28,29]. 

Therefore, the key solution for these problems is the development of nanostructured ZnO by facile synthesis methods. To achieve this target, the growth of nanostructures including nanorods, nanowires, and nanotubes directly on conductive substrates could be considered. Particularly, conductive substrate based electrodes provide excellent electrical contacts, which helps in achieving high electrochemical performance by comparing with the powder electrodes that were coated by a common slurry pasting method [30,31]. 

Until now, ZnO nanorods grow directly on the conductive substrates and are used as binder-free electrode for supercapacitors has not yet been reported. In this work, we have demonstrated, for the very first time, a facile and versatile strategy for the growth of ZnO nanorods vertically on Al substrate by using low temperature (80 °C) wet chemical method for asymmetrical supercapacitors containing a binder-free electrode. The wet chemical method has the advantage of low operating temperatures (below 100 °C), and rapid and low process costs. Electrochemical studies of the ZnO nanorods electrode demonstrated a high value of specific capacitance of 394 F g^−1^ at a scan rate of 20 mV s^−1^, and excellent stability of >98% capacitance maintained even after 1000 cycles at 1 A g^−1^. The presented approach for the growth of ZnO nanostructures might provide a path for the preparation of cost effective binder-free electrode.

## 2. Experimental Details

**Figure 1:**
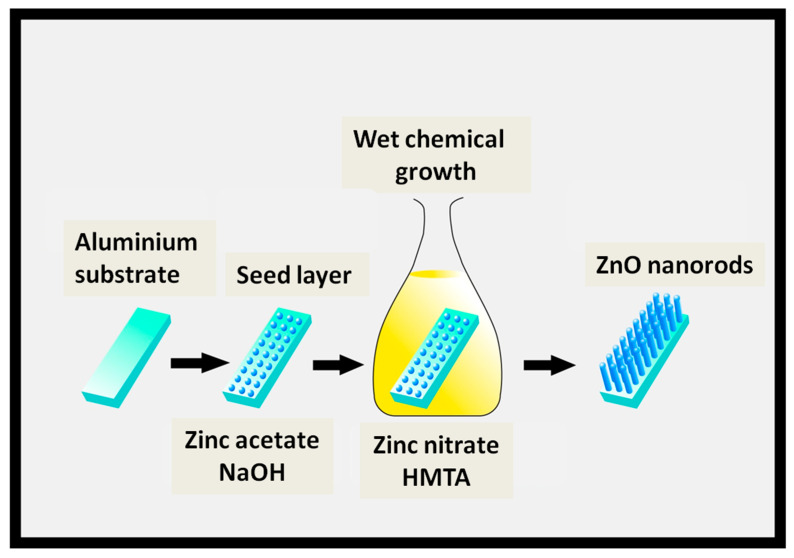
A Schematic diagram for the growth of ZnO nanorods on Al substrate.

ZnO nanorods with high crystalline nature were grown on Al substrate (15 µM thick sheets; MTI, Richmond, CA, USA) that was seeded with ZnO particles. The Al foil was pre-treated prior to the seeding step. The foil was washed by acetone (99.99%), ethanol (99.99%), and distilled water) sequentially. After the cleaning, foil was dried in an oven at 80 °C. The dried Al foil was then used for seeding. The fabrication of ZnO nanorods was performed in two steps. The first step was performed to deposit a seed layer with spin coating using ZnO solution containing a mixture of 30 mM NaOH and 10 mM zinc acetate solution in ethanol, followed by heating at 200 °C for 5 min. The second step resulted in ZnO nanorods growth and was carried out by using the wet chemical method. An equimolar (10 mM) aqueous solution of hexamethylenetetramine (HMT) (C_6_H_12_N_4_; 99.99%, Sigma Aldrich) and zinc nitrate (Zn(NO)_3_·6H_2_O; 99.99%, Sigma Aldrich, St. Louis, MO, USA) was prepared for the growth. For the synthesis, HMT solution was preheated at 80 °C, in which zinc nitrate solution was mixed, and then Al substrate was put into the solution in seed facing down position. This reaction was carried on for 1 h, and vertically aligned ZnO nanorods on Al substrate were grown. Lastly, the washing of as grown ZnO nanorods was performed numerous times in deionized water, followed by drying at 80 °C for 12 h. Figure 1 demonstrates a schematic drawing of the ZnO nanorods growth on Al substrate.

The structural information of the product was obtained using X-ray diffractometer (Phillips X’pert; MPD 3040, EA Almelo, The Netherlands) that was equipped with Cu Kα radiations. Morphological studies of the product were carried out by using field-emission scanning electron microscope (FE-SEM, TESCAN, MIRA II LMH, Brno–Kohoutovice, Czech Republic) and transmission electron microscope (TEM; JEOL 2100, Corporation Place, Singapore). To obtain further structural information of ZnO nanorods, Raman spectrum was recorded with the help of Raman spectrophotometer (NRS-3100, JASCO, MD, USA) with a wavelength of 532 nm. In order to perform optical characterization of ZnO nanorods, a Photo-luminescence (PL) spectrometer (JASCO, FP-6500, MD, USA) was used at room temperature. Electrochemical measurements of the ZnO nanorods were performed using three electrode cells by an electrochemical analyzer system (Gamry 600, PA, USA). For the working electrode, binder-free ZnO nanorods on Al substrate were used. All of the electrochemical studies were carried out in 2 M KOH with a counter electrode of a Pt wire and Ag/AgCl served for the reference electrode. A potential range of −1.6 to 1.0 V was used for the cyclic voltammetry (CV) studies, while Charge–discharge (CD) studies were conducted over 0 to 1.0 V. For the calculation of specific capacitance using the CV and CD method, the following equations have been used [32]:

(1) $$C=\frac{1}{2mVk}\int_{V^{-}}^{V^{+}}I\left(V\right)dV\;\mathrm{By}\;\mathrm{CV}$$

(2) $$C=\frac{I\times \Delta t}{V\times m}\;\mathrm{By}\;\mathrm{CD}$$

where *C* (F g^−1^) defines the specific capacitance, *I* (A) represents discharge current, Δ*t* (s) is discharge time, while *V* (V) corresponds to the potential range, *m* (g) represents the mass used of the active material, and *k* (V s^−1^) is scan rate. A frequency that ranges from 1 Hz–100 kHz was used for the electrochemical impedance spectroscopy (EIS) analysis.

## 3. Results and Discussion

**Figure 2:**
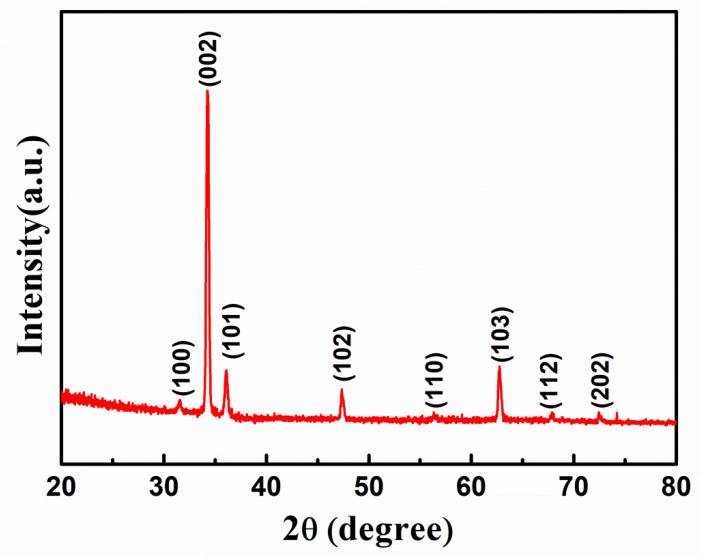
XRD patterns of ZnO nanorods grown on Al substrate.

Figure 2 depicts the XRD patterns of ZnO nanorods grown vertically on Al substrate. Various researchers have used Al_2_O_3_ substrate to grow ZnO nanorods. You et al. [33] reported the growth of ZnO and ZnMgO films on Al_2_O_3_ (001) substrate. They have shown that besides the sapphire substrate diffraction peak located at 41.7°, only (002) and (004) diffraction peaks of ZnO at about 34.3° and 72.4° are observed for the ZnO film, which indicated that the ZnO thin film was grown along a c-axis orientation of the sapphire substrate. Their XRD results indicated that, due to the preferred single orientation (001) of the Al_2_O_3_ substrate, only peaks that are parallel to (001) planes of ZnO are observed in the XRD pattern. While, no other peaks that belong to ZnO can be seen in the XRD pattern. The similar behavior was also observed in another report that was presented by Yang et al. [34] on the growth of ZnO thin films on sapphire (001) substrates. They showed that only two peaks thatcontributed to the (002) and (004) planes of ZnO were observed. No other peaks were detected within the detection limit of XRD, which conclude that ZnO grown on Al_2_O_3_ (001) substrate results in the growth of ZnO along (001) parallel planes only. However, in our work, we have used Al substrate for the growth of ZnO nanorods. Figure S1 shows the XRD pattern of Al substrate used in our work. The diffraction peaks observed at 2θ values of 38.28, 44.59, and 64.91 correspond to the (111), (200), and (220) planes of the bare Al substrate (JCPDF card # 85-1327) [35]. All the peaks in our XRD pattern (Figure 2) are indexed to wurtzite ZnO structure, and in close agreement with the standard data of JCPDS 89-1397. Among all of the planes of ZnO in the XRD patterns, the intensity of (002) plane is extremely high, which clearly reveals that the ZnO nanorods possess single-phase with high crystallinity and grow along the c-axis. There are other peaks present that correspond to ZnO with less intensity than that of (002) plane of ZnO. However, the peak related to Al was not observed within the detection limit of XRD, it might be due to very high intensity of (002) peaks of ZnO, which suppressed other peaks either related to Al or ZnO. Therefore, it is clear that we have used Al substrate for the growth of ZnO nanorods and Al_2_O_3_ was not used. IfAl_2_O_3_ substrate was used in our work, the XRD pattern must have shown only peaks parallel to (001) plane of ZnO along with (006) peak of Al_2_O_3_ substrate, as reported in previous work, but this is not the case in our XRD results. Thus, the possibility of the use of Al_2_O_3_ substrate in this work has been ruled out. Table S1 summarizes the discussion.

**Figure 3:**
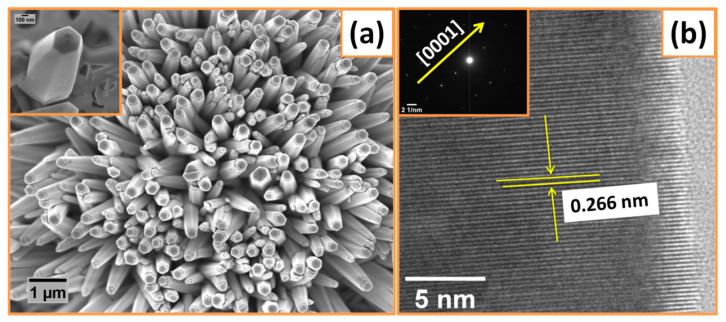
(**a**) Field-emission scanning electron microscope (FE-SEM) image of ZnO nanorods, inset shows a single nanorod. (**b**) HRTEM image, and inset shows the SAED pattern of ZnO nanorods.

Morphological studies of the as-grown ZnO nanorods were performed by FESEM and TEM techniques and are shown in Figure 3. Figure 3a shows the FESEM micrographs of vertically aligned ZnO nanorods that were grown on Al substrate synthesized at 80 °C. The overall morphological features of the ZnO nanorods could be evidently seen and revealed the formation of clear nanorods. The inset of Figure 3a shows that each individual ZnO nanorod consists of a well-defined hexagon facet. The nanorods have length and diameter of ~2 µm and 100–180 nm, respectively, and the size distribution is homogenous throughout the surface of Al substrate. The homogeneity of such nanostructures is helpful in achieving high energy density by facilitating the charge transport during charge and discharge [29,36]. Additionally, a good electrical contact could be assured with the unique design of the electrode with such morphology on Al current collector/substrate; as a result, the area of interaction on the electrode/electrolyte interface might increase.

Additional morphological features and crystalline quality of ZnO nanorods were achieved through TEM investigations. The atomic structure related information obtained from HRTEM image in Figure 3b depicts the highly crystalline nature of ZnO nanorods with interlayer spacing of 0.266 nm, which attributes to the d spacing of (002) lattice plane in ZnO structures. The results that were obtained from HRTEM further confirm the XRD studies where the ZnO nanorods have preferred growth direction along *c*-axis. The inset of Figure 3b shows the SAED patterns of the nanorods containing bright dot patterns that correspond to single-crystal behavior of as-prepared ZnO nanorods. 

**Figure 4:**
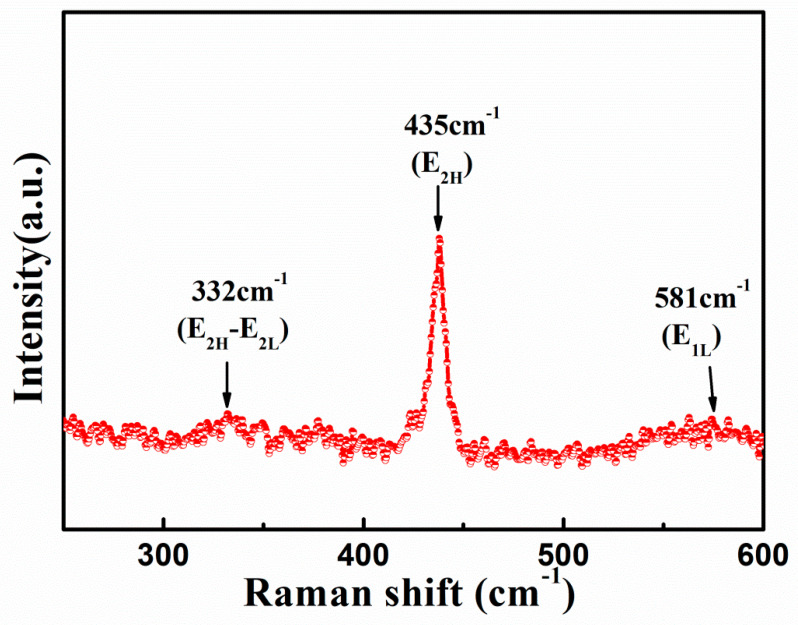
Raman spectrum for ZnO nanorods recorded at room temperature.

Raman spectroscopy was performed at room temperature in order to study the defects and crystal structure of ZnO nanorods. Generally, ZnO with a space group of C^4^_6V_ and wurtzite structure contains two formula units in a primitive cell [37]. The origin of Raman active modes in the spectra are usually governed by various symmetries, and the frequency of vibrations might shift due to the change in chemical surroundings and spacing between lattices [38]. Figure 4 shows room temperature Raman plot of ZnO nanorods with the peaks positioned at 332 cm^−1^, 435 cm^−1^, and 581 cm^−1^. The peak at 435 cm^−1^ corresponds to the characteristics E_2H_ mode of ZnO wurtzite structure. The peaks that were observed at 332 cm^−1^ and 581 cm^−1^ belong to the Raman E_2H_-E_2L_ and E_1L_ mode, respectively, of ZnO. The high intensity of E_2H_ mode as compared to other modes further reveals the *c*-axis orientation of ZnO nanorods.

**Figure 5:**
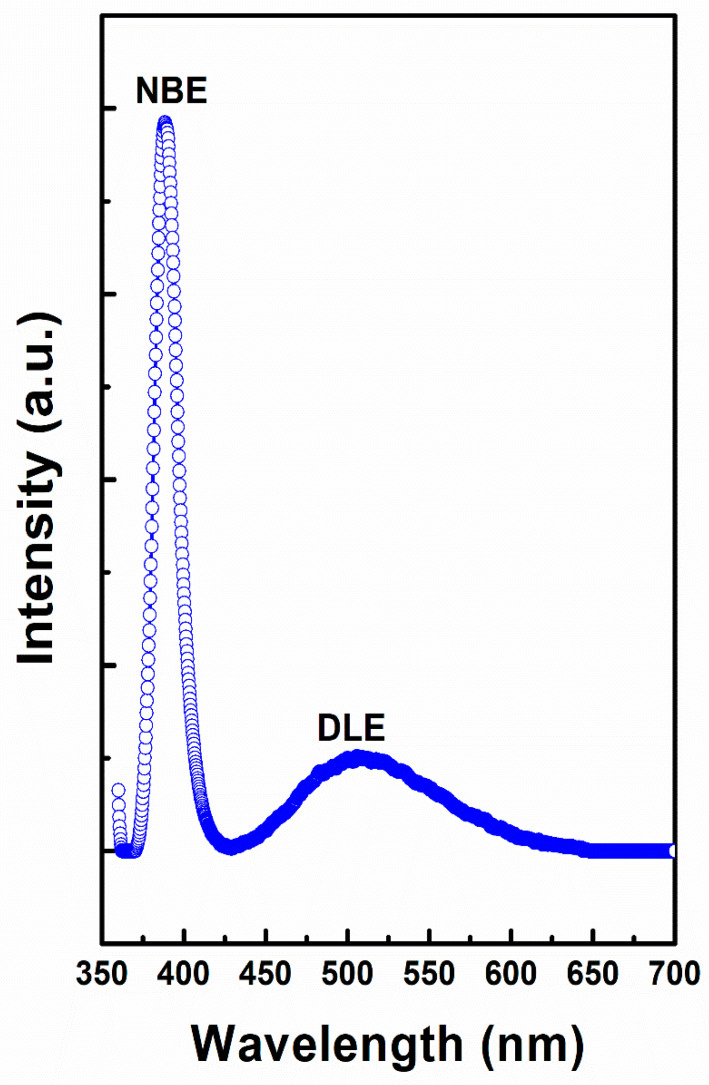
Photoluminescence spectrum of ZnO nanorods at room temperature.

Figure 5 shows the room temperature PL spectrum of the ZnO nanorods. A high intensity near-band-edge (NBE) emission centered at ~388 nm, and a broad peak at ~507 nm (green light) corresponds to deep level emission (DLE) were appeared. The free excitons of ZnO recombined and resulted to the origin of NBE emission, while the photo-originated hole recombination with the defects that were generated green light emission [39,40]. Moreover, the narrow size of full-width at half-maximum (FWHM) of the NBE reveals that ZnO nanorods possess excellent crystalline quality, which further confirms the FESEM study.

**Figure 6:**
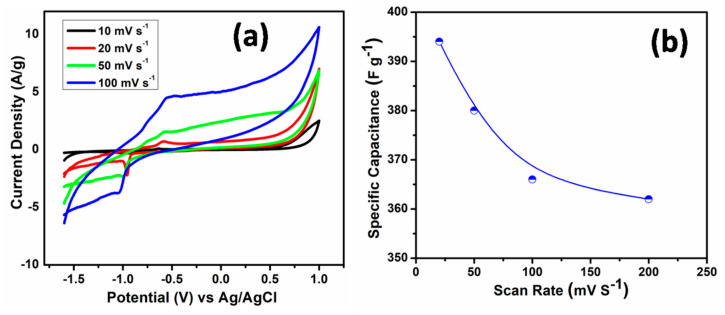
(**a**) CVs of ZnO nanorods electrode at various scan rates with 2M KOH electrolyte and Ag/AgCl as reference electrode. (**b**) Variation of specific capacitance of ZnO nanorods electrode with different scan rate.

Figure 6a reveals the cyclic voltammograms (CV) of ZnO nanorods electrodes in aqueous electrolyte containing 2 M KOH and recorded with various scan rates of 20, 50, 100, and 200 mV s^−1^ in the potential window of −1.6 to +1.0 V vs. Ag/AgCl. It can be clearly seen from CV plot in Figure 6a that by increasing scan rate, the current increases, which depicts the capacitance behavior of the ZnO nanorods. Additionally, as seen in Figure 6a, the CV curves exhibit redox peaks confirming the Faradic nature of the ZnO. The value of specific capacitance of ZnO nanorods electrodes obtained from CV curves was found to be ~394 F g^−1^ at 20 mV s^−1^ scan rate. It was observed that, with increasing in scan rates, the value of capacitance tends to decrease, as shown in Figure 6b. The number of active ions contributed during the redox reaction is the highest in lower scan rates, while it decreased in higher scan rates. In order to make Al substrate stable in KOH electrolyte, on one side of the Al substrate, ZnO was deposited, however, on the other side of Al substrate, a scotch tape layer was used to cover and protect it from being reacted with KOH solution which makes the Al substrate stable in KOH. A CV plot of bare Al substrate (covered with scotch tape on both sides) performed at a scan rate of 20 mV/s is shown in Figure S2. In the CV curve, almost negligible redox peaks of bare aluminum substrate, and much smaller current in the range of µA as compared with those of ZnO nanorods that is in Ampere range, which suggests that the capacitance contribution from the Al substrate is insignificant. Therefore, ZnO nanorods grown on Al substrate have been successfully served as the high performance asymmetrical supercapacitor electrode.

**Figure 7:**
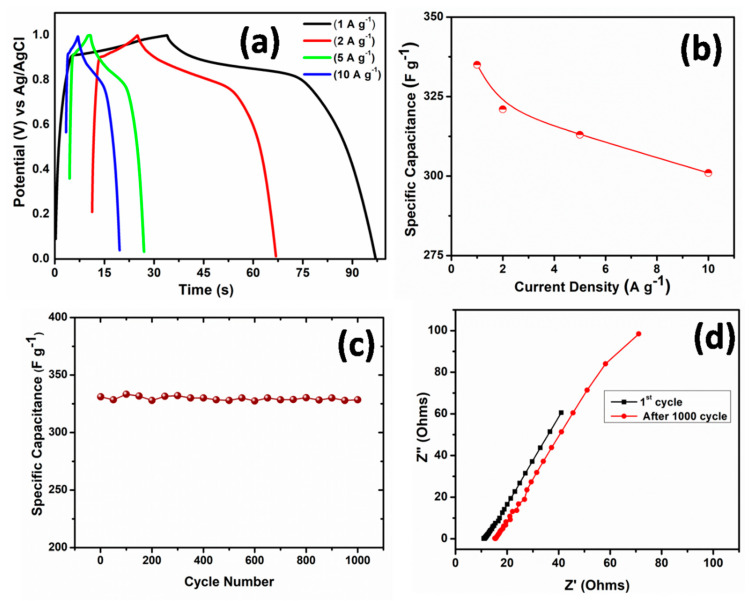
(**a**) Charge-Discharge curves of ZnO nanorods electrode based asymmetrical supercapacitor. (**b**) Variation of specific capacitance of ZnO nanorods electrode with different current density. (**c**) Cyclic performance of ZnO nanorods at a current density of 1 A g^−1^. (**d**) Nyquist plots of ZnO nanorods electrode for 1st and after 1000th cycle.

The charge and discharge characteristics of ZnO nanorods electrode were further confirmed from galvanostatic charge–discharge (CD) curves that were studied at various current densities ranging from 1to 10 A g^−1^, as shown in Figure 7a. A high specific capacitance obtained from charge and discharge curve was found to be ~332 F g^−1^ at a current density of 1 Ag^−1^ and ~301 Fg^−1^ at 10 Ag^−1^. Figure 7b shows the plot of specific capacitance with the change in current density. Interestingly, yet at high value of discharge current density of 10 A g^−1^, there is a small reduction in the specific capacitance depicting excellent rate capacitance features of ZnO nanorods electrode. 

The cycling behavior of the ZnO electrode was performed for 1000 cycles in order to analyze the cyclic stability of the ZnO nanorods electrode, as shown in Figure 7c. Long-term cycling of ZnO nanorods electrode shows no obvious fade, except a very small decrease of ~2% in the specific capacitance, even after 1000 cycles. These cyclic stability results of ZnO nanorods on Al substrate based electrode indicated that minor changes might occur in the physical or chemical structure during the charge discharge cycling procedure. The performance of the ZnO nanorods electrode in terms of specific capacity (mAh/g) vs. cycle number has been studied and shown in Figure S3. It could be clearly seen from Figure S3 that the specific capacity of 58.33 mAh/g was observed for the first cycle, which was maintained for 1000 cycles with capacity retention of ~98%.

The highest specific capacitance of 394 F g^−1^ at a scan rate of 20 mVs^−1^ from CV and 332 F g^−1^ from charge–discharge obtained in our work for ZnO nanorods is superior and highly attractive than that of other ZnO based electrodes that were reported in the literature (see Table 1). He et al. [24] reported a value of 378.5 F g^−1^ for ZnO nanocones and 191.5 F g^−1^ for ZnO NWs. Alver et al. [25] reported specific capacitance of 5.87 F g^−1^, 5.35 F g^−1^ and 4.14 F g^−1^ for ZnO electrodes obtained from nitrate, acetate and chloride precursor solutions, respectively. Luo et al. [26] showed maximum capacitance of 160.4 F g^−1^ for electrodes from ZnO tetrapods. By comparing our results with carbon nanostructures based electrode without metal oxide, it was observed that a capacitance value of 370 F g^−1^ at 1 A g^−1^ in 6 M NaOH for graphene, as reported by Xiao et al. [9], is lower than that of the specific capacitance (394 F g^−1^) of our ZnO electrode. Additionally, the performance of our ZnO nanorods supercapacitors electrode is much better than the transition metal oxides, sulfides, and ZnO based nanocomposites reported earlier [41,42,43,44,45] (see Table 1). In our work, the enhanced performance of ZnO nanorods might be ascribed to the structural advantages of the ZnO on Al substrate assisted prepared electrode.

Electrochemical impedance spectroscopy (EIS) was used to further study the electrochemical characteristics of the ZnO nanorods electrode/electrolyte interface. Figure 7d depicts the typical Nyquist plots of ZnO nanorods electrodes before and after cycling test. In high frequency region, the x-intercept of the Nyquist curve reflects the equivalent series resistance (ESR), which results from the origin of resistance from various sources, such as the electrolyte, internal resistance of electrode material, and the interface resistance [46]. While, the diameter of the semicircle attributes to the resistance of charge transfer on the surface of the electrode (Rct) [47,48]. The value of ESR of ZnO nanorods electrode before cycling test was measured to be 8.1 Ω, and after 1000 cycles, a little increase in the ESR value of about 12.4 Ω was observed, as shown in Figure 7d. This smaller resistance and its minute change even after 1000 cycles might be due to the enhanced contact of the electrode material with the electrolyte. Interestingly, in the Nyquist plot, there was no distinct semicircle observed, which further indicated that ZnO nanorods electrodes exhibit excellent capacitive behavior [49].

## 4. Conclusions

In summary, ZnO nanorods on Al substrate as electrode for supercapacitors have been successfully grown at low temperature of 80 °C while using a facile wet chemical route with excellent electrochemical performance. As grown ZnO nanorods possessed high crystallinity and growth direction along the c-axis which are in close agreement with the HRTEM and SAED results. The FESEM results demonstrate well-defined hexagonal shape of ZnO nanorods homogeneously grown on Al substrate. Raman studies show that a high intensity E_2H_ mode of the wurtzite lattice is evidenced in addition to the low intensity broad peaks of other modes. The PL spectrum demonstrates a strong NBE emission positioned at ~388 nm and a broad DLE emission at ~507 nm. The electrochemical studies demonstrate that the grown ZnO nanorods electrode exhibit superior performance with the specific capacitance of 394 F g^−1^ at 20 mV s^−1^ scan rate and high cyclic efficiencies of >98% after 1000 cycles; thus, it could be considered to bea potential candidate of electrode for supercapacitors. The presented simple and cost effective approach for large scale production of ZnO nanorods on Al substrate is highly attractive and might be applicable for the development of future binder-free metal oxide electrodes for supercapacitors.

## Figures and Tables

**Table 1:** A comparative study of specific capacitance between current work and earlier reports.

Electrode Material	Scan Rate (mV s^−1^)	Specific Capacitance (F g^−1^)	Ref.
ZnO nanocones	20	377.4	[24]
ZnO nanostructures	5	5.87	[25]
ZnO tetrapods	10	160.4	[26]
ZnO coated with MnO_2_	25	222	[16]
ZnO/RGO	5	322.1	[41]
CeO_2_/Fe_2_O_3_ nanospindles	5	142.6	[42]
MoS_2_ on Mo foil	5	197.1	[43]
CoMoO_4_	5	98.34	[44]
ZnS/graphene	5	197.1	[45]
ZnO nanorods on Al substrate	20	394.1	This work

## Supplementary Materials

The following are available online at https://www.mdpi.com/2079-4991/10/10/1979/s1, Figure S1: XRD pattern of Al substrate used in the present work., Figure S2: CV plot of bare Al substrate (covered with scotch tape) in 2M KOH electrolyte, Figure S3: Plot of Specific capacity vs cycle number for ZnO nanorods electrode at current density of 1 A/g, Table S1: Comparison of ZnO grown on Al and Al_2_O_3_ substrate.

## References

[1] Shao Y.L., El-Kady M.F., Wang L.J., Zhang Q.H., Li Y.G., Wang H.Z., Mousavi M.F., Kaner R.B. (2015). Graphene-based materials for flexible supercapacitors. Chem. Soc. Rev..

[2] Zhong C., Deng Y.D., Hu W.B., Qiao J.L., Zhang L., Zhang J.J. (2015). A review of electrolyte materials and compositions for electrochemical supercapacitors. Chem. Soc. Rev..

[3] Liang J., Zhu G., Lu Z., Zhao P., Wang C., Ma Y., Xu Z., Wang Y., Hu Y., Ma L. (2018). Integrated perovskite solar capacitors with high energy conversion efficiency and fast photo-charging rate. J. Mater. Chem. A.

[4] Simon P., Gogotsi Y. (2008). Materials for electrochemical capacitors. Nat. Mater..

[5] Bagheri N., Aghaei A., Ghotbi M.Y., Marzbanrad E., Vlachopoulos N., Häggman L., Wang M., Boschloo G., Hagfeldt A., Skunik-Nuckowska M. (2014). Combination of asymmetric supercapacitor utilizing activated carbon and nickel oxide with cobalt polypyridyl-based dye-sensitized solar cell. Electrochim. Acta.

[6] Navarrete-Astorga E., Rodr´ıguez-Moreno J., Dalchiele E.A., Schrebler R., Leyton P., Ramos-Barrado J.R., Martín F. (2017). A transparent solid-state ion gel for supercapacitor device applications. J. Solid State Electrochem..

[7] Rodríguez-Moreno J., Navarrete-Astorga E., Dalchiele E.A., Sánchez L., Ramos-Barrado J.R., Martín F. (2013). Polyvinylpyrrolidone–LiClO_4_ solid polymer electrolyte and its application in transparent thin film supercapacitors. J. Power Sources.

[8] Rodríguez-Moreno J., Navarrete-Astorga E., Dalchiele E.A., Schrebler R., Ramos-Barrado J.R., Martín F. (2014). Vertically aligned ZnO@CuS@PEDOTcore@shellnanorod arrays decorated with MnO_2_ nanoparticles for a high-performance and semi-transparent supercapacitor electrode. Chem. Commun..

[9] Xiao N., Tan H., Zhu J., Tan L., Rui X., Dong X., Yan Q. (2013). High-Performance Supercapacitor Electrodes Based on Graphene Achieved by Thermal Treatment with the Aid of Nitric Acid. ACS Appl. Mater. Interface.

[10] Hu C.C., Chang K.H., Lin M.C., Wu Y.T. (2006). Design and tailoring of the nanotubular arrayed architecture of hydrous RuO_2_ for next generation supercapacitors. Nano Lett..

[11] Hung C.J., Hung J.H., Lin P., Tseng T.Y. (2011). Electrophoretic fabrication and characterizations of manganese oxide/carbon nanotube nanocomposite pseudocapacitors. J. Electrochem. Soc..

[12] Chen Z., Augustyn V., Wen J., Zhang Y., Shen M., Dunn B., Lu Y. (2011). High-performance supercapacitors based on intertwined CNT/V_2_O_5_ nanowire nanocomposites. Adv. Mater..

[13] Kalpana D., Omkumar K.S., Kumar S.S., Renganathan N.G. (2006). A novel high power symmetric ZnO/carbon aerogel composite electrode for electrochemical supercapacitor. Electrochim. Acta.

[14] Kim I.H., Kim K.B. (2006). Electrochemical characterization of hydrous ruthenium oxide thin-film electrodes for electrochemical capacitor applications. J. Electrochem. Soc..

[15] Li X., Wang Z., Qiu Y., Pan Q., Hu P.A. (2015). 3D Graphene/ZnONanorods Composite Networks as Supercapacitor Electrodes. J. Alloys Compd..

[16] Huang G., Zhang W., Xu S., Li Y., Yang Y. (2016). MicrosphericalZnO Synthesized from a Metal-Organic Precursor for Supercapacitors. Ionics.

[17] Chen H.C., Lyu Y.R., Fang A., Lee G.J., Karuppasamy L., Wu J.J., Lin C.K., Anandan S., Chen C.Y. (2020). The Design of ZnONanorod Arrays Coated with MnOx for High Electrochemical Stability of a Pseudocapacitor Electrode. Nanomaterials.

[18] Gao J.W.Z., Li Z., Wang B., Yan Y., Liu Q., Mann T., Zhang M., Jiang Z. (2011). Green synthesis of graphene nanosheets/ZnO composites and electrochemical properties. J Solid State Chem..

[19] Lu T., Pan L., Li H., Zhu G., Lv T., Liu X., Sun Z., Chen T., Chua D.H. (2011). Microwave-assisted synthesis of graphene-ZnO nanocomposites for electrochemical supercapacitors. J. Alloy Compd..

[20] Zhang Y., Sun X., Pan L. (2009). Carbon nanotube–ZnO nanocomposite electrodes for supercapacitors. Solid State Ion..

[21] Zhang Y., Li H., Lu T. (2009). Capacitive behavior of graphene–ZnO composite film for supercapacitors. J. Electroanal. Chem..

[22] Jayalakshmi M., Palaniappa M., Balasubramanian K. (2008). Single step solution combustion synthesis of ZnO/carbon composite and its electrochemical characterization for supercapacitor application. Int. J. Electrochem. Sci..

[23] Kim C.H., Kim B.H. (2015). Zinc oxide/activated carbon nanofiber composites for high-performance supercapacitor electrodes. J. Power Sources.

[24] He X., Yoo J.E., Lee M.H., Bae J. (2017). Morphology Engineering of ZnO Nanostructures for High Performance Supercapacitors: Enhanced Electrochemistry of ZnONanocones Compared to ZnO Nanowires. Nanotechnology.

[25] Alver Ü., Tanrıverdi A., Akgül Ö. (2016). Hydrothermal Preparation of ZnO Electrodes Synthesized from Different Precursors for Electrochemical Supercapacitors. Synth. Met..

[26] Luo Q., Xu P., Qiu Y., Cheng Z., Chang X., Fan H. (2017). Synthesis of ZnO Tetrapods for High-Performance Supercapacitor Applications. Mater. Lett..

[27] Fu Z.W., Feng H., Ye Z., Yue C., Qin Q.Z. (2003). The electrochemical reaction of zinc oxide thin films with lithium. J. Electrochem. Soc..

[28] Woo M.A., Kim T.W., Kim I.Y., Hwang S.J. (2011). Synthesis and lithium electrode application of ZnO-ZnFe_2_O_4_ nanocomposites and porously assembled ZnFe_2_O_4_ nanoparticles. Solid State Ion..

[29] Park K.T., Xia F., Kim S.W., Kim S.B., Song T., Paik U., Park W.I. (2013). Facile synthesis of ultrathin ZnO nanotubes with wellorganized hexagonal nanowalls and sealed layouts: Applications for lithium ion battery anodes. J. Phys. Chem. C.

[30] Greene L.E., Law M., Tan D.H., Montano M., Goldberger J., Somorjai G., Yang P.D. (2005). General Route to Vertical ZnO Nanowire Arrays Using Textured ZnO Seeds. Nano Lett..

[31] Wang Y.Y., Jiang X.J., Yang L.S., Jia N., Ding Y. (2014). In situ synthesis of C/Cu/ZnO porous hybrids as anode materials for lithium ion batteries. ACS Appl. Mater. Interfaces.

[32] Chen J., Liu Y., Li W., Yang H., Xu L. (2015). The large electrochemical capacitance of nitrogen doped mesoporous carbon derived from egg white by using a ZnO template. RSC Adv..

[33] You J.B., Zhang X.W., Dong J.J., Song X.M., Yin Z.G., Chen N.F., Yan H. (2009). Localized-Surface-Plasmon Enhanced the 357 nm Forward Emission from ZnMgO Films Capped by Pt Nanoparticles. Nanoscale Res. Lett..

[34] Yang W., Wang F., Guan Z., He P., Liu Z., Hu L., Chen M., Zhang C., He X., Fu Y. (2019). Comparative Study of ZnO Thin Films Grown on Quartz Glass and Sapphire (001) Substrates by Means of Magnetron Sputtering and High-Temperature Annealing. Appl. Sci..

[35] Gaddama V., Neellaa N., Nayakb M.M., Rajannaa K. (2018). Al:ZnONanosheets on Flexible Stainless Steel Substrate as Impact Sensor. Mater. Today Proc..

[36] Zhao S.H., Guo J.X., Jiang F., Su Q.M., Zhang J., Du G.H. (2016). Growth of hierarchal porous CoO nanowire arrays on carbon cloth as binder-free anodes for high-performance flexible lithium-ion batteries. J. Alloys Compd..

[37] Arguello C.A., Rousseau D.L., Porto S.P.S. (1969). First-order Raman effect in wurtzite-type crystals. Phys. Rev..

[38] Damen T.C., Porto S.P.S., Tell B. (1966). Raman effect in zinc oxide. Phys. Rev..

[39] Huang M.H., Wu Y., Feick H., Tran N., Weber E., Yang P. (2001). Catalytic growth of zinc oxide nanowires by vapor transport. Adv. Mater..

[40] Hung C.H., Whang W.T. (2003). A novel low-temperature growth and characterization of single crystal ZnO nanorods. Mater. Chem. Phys..

[41] Hwang Y.H., Lee S.M., Kim Y.J., Kahng Y.H., Lee K. (2016). A new approach of structural and chemical modification on graphene electrodes for high-performance supercapacitors. Carbon.

[42] Arul N.S., Mangalaraj D., Ramachandran R., Grace A.N., Han J.I. (2015). Fabrication of CeO_2_/Fe_2_O_3_ composite nanospindles for enchancedvisble light driven photocatalysts and supercapacitor electrodes. J. Mater. Chem. A.

[43] Krishnamoorthy K., Veerasubramani G.K., Pazhamalai P., Kim S.J. (2016). Designing two dimensional nanoarchitectured MoS_2_ sheets grown on Mo foil as a binder free electrode for supercapacitors. Electrochim. Acta.

[44] GVeerasubramani K., Krishnamoorthy K., Kim S.J. (2015). Electrochemical performance of an asymmetric supercapacitor based on graphene and cobalt molybdate electrodes. RSC Adv..

[45] Ramachandran R., Saranya M., Kollu P., Raghupathy B.P.C., Jeong S.K., Gracem A.N. (2015). Solvothermal synthesis of zinc sulfide decorated graphene (ZnS/G) nanocomposites for novel supercapacitor electrodes. Electrochim. Acta.

[46] Zhang J., Kong L.B., Cai J.J., Luo Y.C., Kang L. (2010). Nanoflake-like cobalt hydroxide/ ordered mesoporous carbon composite for electrochemical capacitors. J. Solid State Electrochem..

[47] Shi S., Zhuang X., Chen B., Wang X. (2013). Solution blowig of ZnO nanoflake-encapsulated carbon nanofibers as electrodes for supercapacitors. J. Mater. Chem. A.

[48] Zhang X., Wang X., Jiang L., Wu H., Wu C., Su J. (2012). Effect of aqueous electrolytes on the electrochemical behaviors of supercapacitors based on hierarchically porous carbons. J. Power Sources.

[49] Niu H., Zhou D., Yang X., Li X., Wang Q., Qu F.Y. (2015). Towards three-dimensional hierarchical ZnO nanofiber@Ni(OH)_2_nanoflake core–shell heterostructures for high performance asymmetric supercapacitors. J. Mater. Chem. A.

