# Supplementary material for: Binder-Free Electrode Based on ZnO Nanorods Directly Grown on Aluminum Substrate for High Performance Supercapacitors
Source: Nanomaterials (Basel). 2020 Oct 7;10(10):1979. doi: 10.3390/nano10101979 (PMC7599740; doi:10.3390/nano10101979)
Supplement: Supplementary file 1 [file nanomaterials-10-01979-s001.pdf]

Supplementary Materials

Binder-Free Electrode Based on ZnONanorods Directly Grown on Aluminum Substrate for High Performance Supercapacitors

Faheem Ahmed [1,*], Ghzzai Almutairi [2,*], Bandar AlOtaibi [2], Shalendra Kumar [1], NishatArshi [3], Syed Ghazanfar Hussain [1,*], Ahmad Umar [4,5], Naushad Ahmad [6], and Abdullah Aljaafari [1]

1. Department of Physics, College of Science, King Faisal University, P.O. Box-400, Al-Ahsa 31982, Saudi Arabia; sjagdish@kfu.edu.sa (S.K.); aaljaafari@kfu.edu.sa (A.A.)
2. National Center for Energy Storage Technologies, King Abdulaziz City for Science and Technology (KACST), Riyadh 12354, Saudi Arabia; bmalotaibi@kacst.edu.sa
3. Department of Basic Sciences, Preparatory Year Deanship, King Faisal University, Al-Ahsa31982, Saudi Arabia; nshastri@kfu.edu.sa;
4. Department of Chemistry, Faculty of Science and Arts,NajranUniversity, Najran11001, Saudi Arabia; ahmadumar786@gmail.com
5. Promising Centre for Sensors and Electronic Devices (PCSED), Najran University, Najran-11001, Kingdom of Saudi Arabia
6. Department of Chemistry, College of Science, King Saud University, Riyadh 11451, Saudi Arabia; anaushad@ksu.edu.sa

* Correspondence: fahmed@kfu.edu.sa (F.A.); gmotari@kacst.edu.sa (G.A.); sghazanfar@kfu.edu.sa (S.G.H.); Tel./Fax: +966-013589-6900(F.A.)

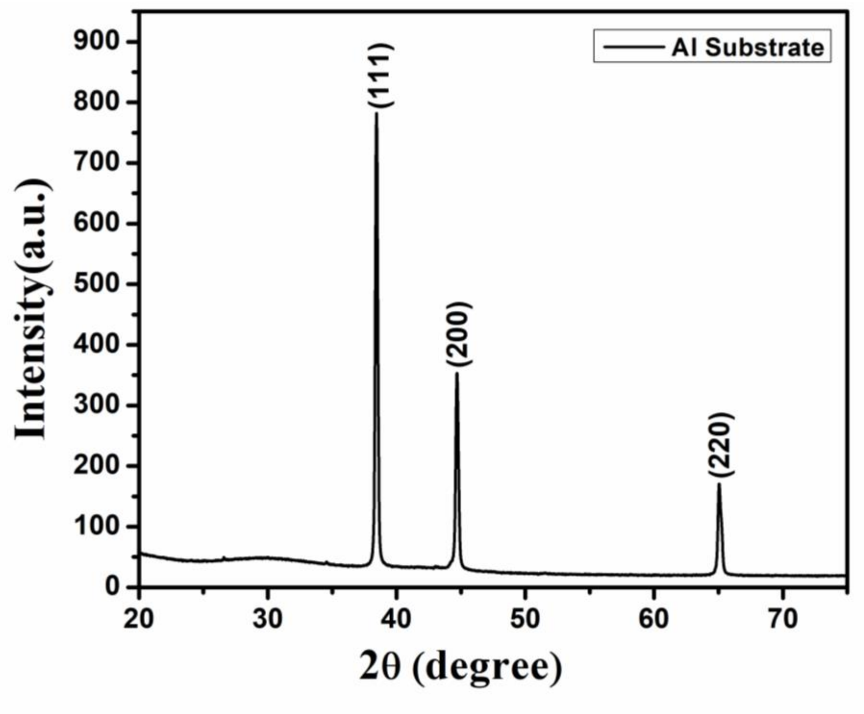

Figure S1. XRD pattern of Al substrate used in the present work.

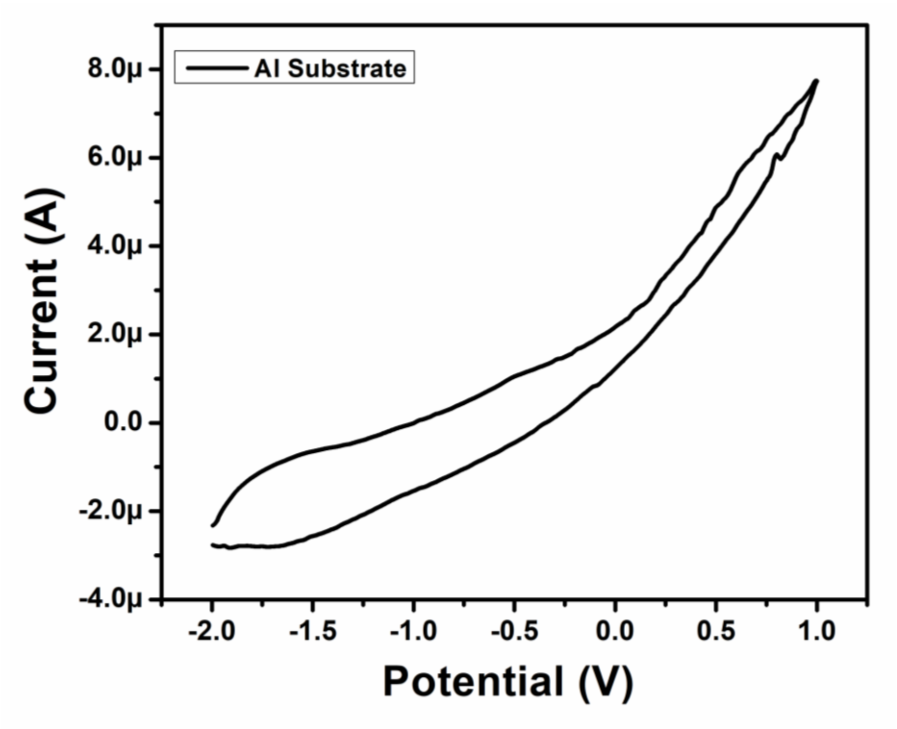

Figure S2. CV plot of bare Al substrate (covered with scotch tape) in 2M KOH electrolyte.

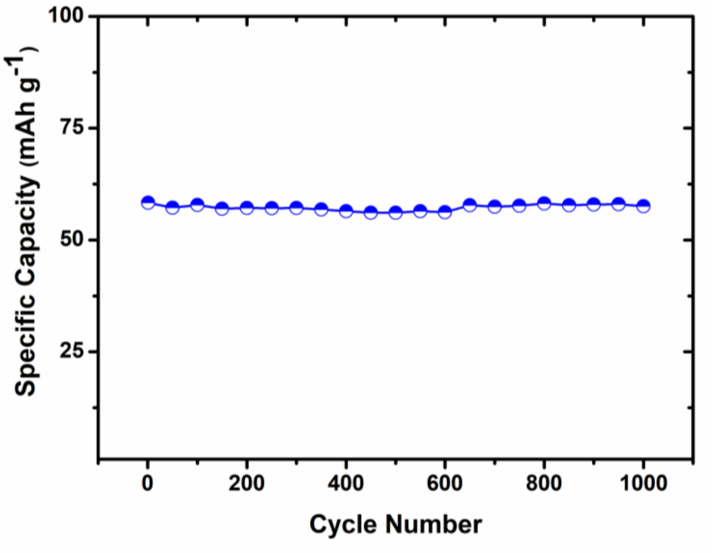

Figure S3. Plot of Specific capacity vs cycle number for ZnO nanorods electrode at current density of 1 A/g.

Table S1. Comparison of ZnO grown on Al and Al_2O_3 substrate.

Substrate	Results (XRD)	Reference

<table>
<tr><td>Al_2O_3 (001) substrate</td><td>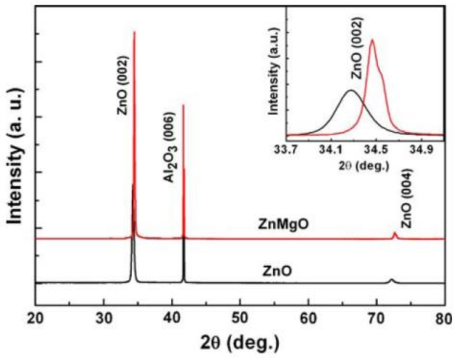
</td><td>[1] J. B. You, X. W. Zhang, J. J. Dong, X. M. Song, Z. G. Yin, N. F. Chen, H. Yan, Nanoscale Res Lett (2009) 4:1121–1125.</td></tr>
<tr><td>Al_2O_3 (001) substrate</td><td>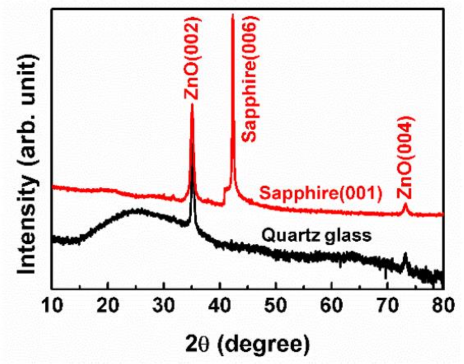
</td><td>[2] Weijia Yang, Fengming Wang, Zeyi Guan, Pengyu He, Zhihao Liu, Linshun Hu,
Mei Chen, Chi Zhang, Xin He, Yuechun Fu, Appl. Sci. 2019, 9, 4509.</td></tr>
<tr><td>Al substrate</td><td>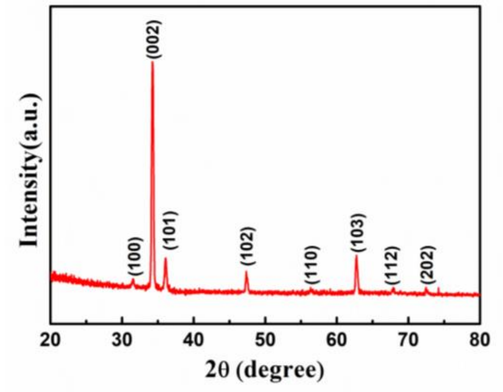
</td><td>This work</td></tr>
</table>
